# COVID Vaccine Rollout for Older Adults in France

**DOI:** 10.1093/geroni/igab046.715

**Published:** 2021-12-17

**Authors:** Matthieu De Stampa

**Affiliations:** Assistance Publique - Hôpitaux de Paris, Paris, Ile-de-France, France

## Abstract

Only about three million people in France have so far received at least one dose of a Covid-19 vaccine. Those aged over 75 are offered either Pfizer or Moderna vaccines in a vaccination center. Older people with pre-existing conditions can now get AstraZeneca's Covid-19 vaccine. We will provide detailed information later. The symposium has experts from 8 countries. We will use an interview and dialog format, instead of presentations (please refer to the program overview).

